# OrchAlign: orchestrated multimodal alignment for gene expression prediction

**DOI:** 10.1093/bioinformatics/btag711

**Published:** 2026-09-29

**Authors:** Li Liu, Guipeng Xv, Shujie Liu, Yeyun Gong, Chen Lin

**Affiliations:** School of Informatics, National Institute for Data Science in Health and Medicine, Xiamen University, Xiamen 361005, China; School of Information Engineering, Sanming University, Sanming 365004, China; School of Informatics, National Institute for Data Science in Health and Medicine, Xiamen University, Xiamen 361005, China; Microsoft Research Asia, Microsoft, Beijing 100080, China; Microsoft Research Asia, Microsoft, Beijing 100080, China; School of Informatics, National Institute for Data Science in Health and Medicine, Xiamen University, Xiamen 361005, China

## Abstract

**Motivation:**

Gene expression prediction benefits from integrating DNA sequences and epigenomic signals. Existing approaches typically combine these modalities using simple operations such as concatenation or summation, without explicitly modeling fine-grained token-level cross-modal interactions. Establishing precise cross-modal correspondences remains challenging due to (1) inter-modal discrepancies, and (2) intra-modal structural preservation.

**Results:**

To address these challenges, we propose OrchAlign, an orchestrated multimodal alignment framework that establishes fine-grained cross-modal alignment while preserving intra-modal structure. Our approach first disentangles each modality into shared and specific representations. We then perform focused cross-modal alignment on the shared subspace within a key regulatory region, producing aligned representations with token-level correspondences. Moreover, we enforce consistency between relational structures of the aligned shared representations and their corresponding modality-specific counterparts, enhancing intra-modal structural coherence. Finally, the aligned shared representations and modality-specific representations are passed to the BiMamba backbone for further multimodal fusion. Our experimental results show that OrchAlign achieves state-of-the-art performance in gene expression prediction.

**Availability and implementation:**

All resources are available at https://github.com/XMUDM/OrchAlign

## 1 Introduction

Gene expression is a fundamental biological process through which genetic information encoded in DNA is transformed into functional molecules such as RNA and proteins (San Segundo-Val and Sanz-Lozano 2016). Therefore, gene expression prediction has become a critical task in genomics. Its core objective is to take DNA sequences as input and predict a real-valued output that reflects the expression level of a gene (Beer and Tavazoie 2004, MacNeil and Walhout 2011, Pipoli *et al*. 2022).

Previous approaches rely solely on DNA sequences to predict gene expression using sequence models such as transformer-based models (Avsec *et al*. 2021, Pipoli *et al*. 2022) or state space models (Nguyen *et al*. 2023, Gu and Dao 2024, Schiff *et al*. 2024). Given that DNA sequences and epigenomic signals provide complementary views of gene regulation, with the former encoding the static regulatory potential and the latter capturing the dynamic, cell-type-specific epigenetic states, recent methods (Karbalayghareh *et al*. 2022, Lin *et al*. 2024, Wall *et al*. 2024, Su *et al*. 2025) therefore integrate epigenomic signals such as chromatin accessibility with DNA sequences by concatenating them at corresponding genomic positions at the input stage, and then feeding into sequence models, leaving cross-modal relationships to be learned implicitly.

However, such implicit learning does not explicitly model the fact that epigenomic signals are aggregated from sequencing reads across local genomic regions rather than measured independently at each base (Johnson *et al*. 2007).

This aggregation induces a token-level correspondence between epigenomic signals and DNA tokens, where each signal spans multiple DNA bases and each base may contribute to multiple signals. Early input-level concatenation without explicit alignment cannot properly capture these fine-grained cross-modal correspondences.

Therefore, explicit token-level cross-modal alignment is essential for modeling gene regulatory mechanisms underlying gene expression. It enables fine-grained correspondences between DNA sequences and their surrounding epigenomic context, faithfully capturing local regulatory interactions while preserving modality-specific information. Effective token-level cross-modal alignment in genomic contexts poses two key challenges.

### 1.1 Challenge 1: inter-modal discrepancies

DNA sequences and epigenomic signals exhibit profound heterogeneity at both the representational and biological levels, manifested as: (1) an *encoding gap* between discrete DNA tokens and continuous epigenomic signals, and (2) a *regulatory granularity mismatch* between fine-grained DNA regulatory elements (e.g. transcription factor binding motifs) and region-level chromatin state profiles. These divergences make it particularly difficult to establish accurate token-level semantic correspondence between DNA and epigenomic data. This challenge is further exacerbated in *long genomic contexts*, where vast background regions overwhelm sparse regulatory signals, increasing the risk of spurious cross-modal associations.

### 1.2 Challenge 2: intra-modal structural preservation

Each modality contains inherent regulatory structures spanning both intra- and inter-element relationships, reflecting organized biological patterns within DNA sequences and epigenomic profiles. Cross-modal alignment aims to capture shared regulatory information, but indiscriminate alignment may overemphasize shared signals, thereby suppressing modality-specific features and disrupting intra-modal relational structures. This creates a fundamental trade-off between cross-modal correspondence modeling and intra-modal structural integrity.

To address these challenges, we propose *OrchAlign*, a novel three-stage design that establishes reliable inter-modal correspondences while preserving intra-modal structure.

(1) *Pre-Alignment*. We independently encode each modality and disentangle the resulting representations into shared components forming a shared semantic space for cross-modal alignment, and modality-specific components for preserving unique information. (2) *Cross-modal Alignment*. To mitigate spurious associations arising from sparse regulatory signals amidst abundant background regions, we restrict cross-modal alignment to transcription start site (TSS)-centered regions, where regulatory signals are enriched. Within this region, we apply entropic-regularized optimal transport in the shared subspace to construct reliable cross-modal correspondences, which are then used to derive cross-modal mapped representations. We design a *Correspondence-guided* gated fusion mechanism to adaptively integrate shared features and mapped representations. (3) *Post-Alignment*. To further preserve intra-modal structural consistency, we encourage structural consistency in modality-specific representations via the relational structure of the shared space, and then aggregate all representations for multimodal fusion.

Our key contributions are as follows:

- We propose *OrchAlign, the first multimodal alignment framework for gene expression prediction* that explicitly establishes fine-grained cross-modal alignment between DNA sequences and epigenomic signals while preserving intra-modal structural organization, enabling principled multimodal fusion.
- We introduce a focused cross-modal alignment module that models robust correspondences under sparse regulatory signals and enables correspondence-guided gated integration, generating aligned shared representations.
- Extensive experiments across cell lines demonstrate that *OrchAlign* consistently outperforms the strongest baseline with substantial improvements in prediction performance, and comprehensive ablation studies and visualizations validate the effectiveness of each module.

## 2 Materials and methods

### 2.1 Problem formulation

Given a target gene with its DNA sequence and epigenomic signals, let $X=[x_{1},\ldots ,x_{L}]$ denote the one-hot encoded DNA sequence of length *L* over the nucleotide alphabet {A,T,C,G}, where $x_{i}\in R^{4}$, and $E=[e_{1},\ldots ,e_{L}]$ denote the corresponding epigenomic signals, where $e_{i}\in R^{n}$ represents *n* epigenomic measurements, the objective is to predict its expression level $\hat{y}\in R$ based on *X* and *E* to approximate its true expression level y∈R. The input sequence is centered at the transcription start site (TSS) of the gene, i.e. TSS corresponds to the central index L/2.

### 2.2 Overview of OrchAlign

The proposed *OrchAlign* follows a three-stage design consisting of pre-alignment, cross-modal alignment, and post-alignment, as illustrated in Fig. 1. In the *pre-alignment* stage, to bridge the inter-modal discrepancy and provide a clean subspace for alignment, each modality is encoded and disentangled into shared and specific components. The *cross-modal alignment* stage applies optimal transport and gated fusion to align the shared representations in the central regulatory region, thereby capturing many-to-many cross-modal correspondences while suppressing background noise. The *post-alignment* stage aligns the relational structure of the modality-specific representations to that of the aligned shared space, preserving intra-modal structure without suppressing specificity, and then fuses all features to predict the expression level. This design ensures both cross-modal alignment and intra-modal coherence for accurate prediction.

**Figure 1:**
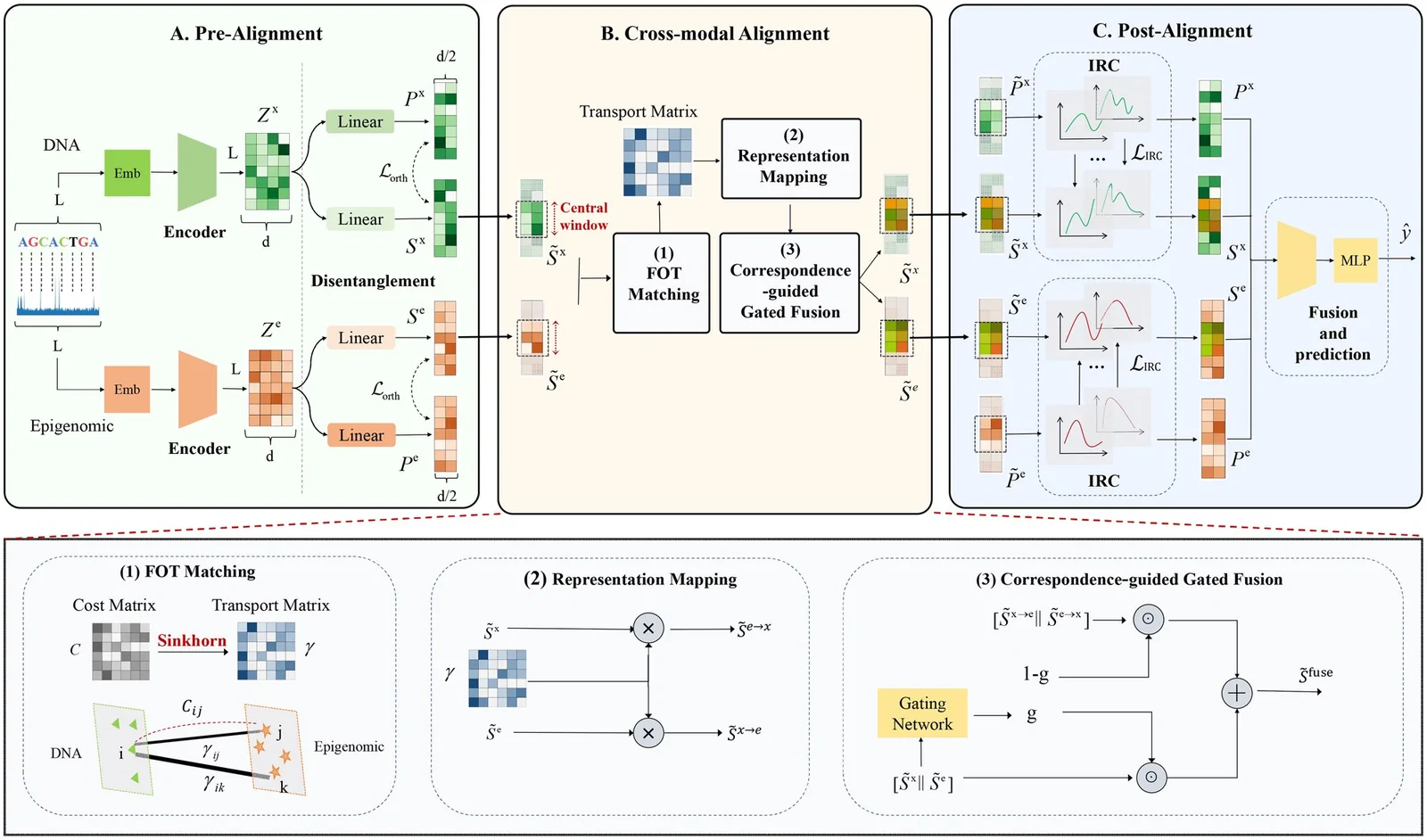
The overview of *OrchAlign*, which follows a three-stage design. (A) *Pre-Alignment* aims to establish a common representational foundation via modality encoding and disentanglement for cross-modal alignment. (B) *Cross-modal Alignment* improves inter-modal consistency in the shared subspace through three steps: (1) focused optimal transport (FOT) for learning token-level correspondences, (2) representation mapping using the learned transport plan, and (3) correspondence-guided gated fusion for integrating original and cross-modal features. (C) *Post-Alignment* enhances intra-modal consistency via an IRC module and integrates multimodal features through a fusion module, producing a unified representation for prediction.

### 2.3 Pre-alignment

To enable effective cross-modal alignment, we must first establish a common representational foundation that reconciles the fundamental heterogeneity between discrete DNA sequences and continuous epigenomic signals. This process involves two steps: *modality-specific encoding followed by feature disentanglement*, which begins by projecting the raw, heterogeneous inputs into an initial embedding space $R^{d}$.


(1) $${\text{Emb}}^{x}=XW^{x},\;{\text{Emb}}^{e}=EW^{e},$$

where $W^{x}\in R^{4\times d},W^{e}\in R^{n\times d}$ are projection matrices.

#### 2.3.1 Unimodal encoders

Inspired by (Su *et al*. 2025), we employ BiMamba (Schiff *et al*. 2024) architecture to encode each modality into context-aware token representations. We deliberately use a shallow configuration with few layers to provide sufficient contextual information while preserving token-level discriminability.


(2) $$Z^{x}=\text{BiMamba}({\text{Emb}}^{x}),\;Z^{e}=\text{BiMamba}({\text{Emb}}^{e}),$$

where $Z^{x},Z^{e}\in R^{L\times d}$ are the outputs of the DNA and epigenomic encoders, respectively.

#### 2.3.2 Feature disentanglement

The encoded DNA sequence and epigenomic features contain both shared regulatory patterns (e.g. transcription factor binding manifested as a sequence motif and a localized epigenomic peak (Mundade *et al*. 2014)) and modality-specific information (e.g. DNA-specific structural features such as CpG Islands (Singh and Sophiarani 2020)). Therefore, to provide a cleaner shared feature space for subsequent cross-modal alignment while preserving modality-specific information, we decouple each modality’s feature into two orthogonal components via a linear layer: modality-*S*hared (*S*) and modality-s *P*ecific (*P*) components.


(3) $$\begin{array}{c} S^{x}=Z^{x}W^{x\to s},\;P^{x}=Z^{x}W^{x\to p},\\ S^{e}=Z^{e}W^{e\to s},\;P^{e}=Z^{e}W^{e\to p}, \end{array}$$

where $W^{x\to s},W^{x\to p},W^{e\to s},W^{e\to p}\in R^{d\times (d/2)}$ are projection matrices. This yields the shared components $S^{x},S^{e}$ and the modality-specific components $P^{x},P^{e}$, each of dimension $R^{L\times (d/2)}$. To promote disentanglement, we introduce an orthogonal constraint, achieved by minimizing an orthogonal loss:


(4) $$L_{\text{orth}}=\sum_{m\in \{x,e\}}\frac{1}{L}\sum_{i=1}^{L}\frac{{\langle S_{i}^{m},P_{i}^{m}\rangle }^{2}}{{\left\|S_{i}^{m}\right\|}_{2}^{2}{\left\|P_{i}^{m}\right\|}_{2}^{2}},$$

where *i* is the token index, *L* is the sequence length, *m* denotes the two modalities, and 〈·,·〉 denotes the inner product operation.

### 2.4 Cross-modal alignment

Given the shared components from the pre-alignment stage, we perform explicit token-level cross-modal alignment to enhance inter-modal consistency by establishing fine-grained regulatory correspondences between DNA tokens and epigenomic signals. To address the sparsity and background dominance of epigenomic signals, we introduce Focused optimal transport (FOT): spatial concentration on the transcription start site (TSS) region, followed by entropic-regularized optimal transport. From the resulting transport plan, we obtain cross-modal mapped representations and adaptively fuse them with the original features via a gated mechanism to produce the final aligned representations.

#### 2.4.1 Focused optimal transport (FOT) matching

The *spatially concentrated* strategy is to focus the alignment on the most critical regulatory region, a subsequence centered on the TSS, where the regulatory landscape is most directly predictive of transcriptional output (Segal *et al*. 2002).

Formally, let *l* denote the length of a TSS-centered region, the index set of this region is defined as $I=\{\frac{L}{2}-\frac{l}{2},\ldots ,\frac{L}{2}+\frac{l}{2}-1\}$. We then obtain the corresponding representations for each modality as:


(5) $$\begin{array}{c} {\tilde{S}}^{x}=\{S_{i}^{x}|i\in I\},\;{\tilde{S}}^{e}=\{S_{i}^{e}|i\in I\}, \end{array}$$

Here, $S_{i}^{x}$ and $S_{i}^{e}$ are the shared representations of two modalities at token position *i*. Within this focused region, to highlight high-confidence cross-modal regulatory links, we further formulate the token-level alignment as an *entropy-regularized Optimal Transport problem*, yielding a sparse and precise matching plan. This problem seeks a transport plan γ by minimizing the regularized transportation cost, formulated as:


(6) $$\begin{array}{cc} & \underset{\gamma \in \Pi (a,b)}{\min}\sum_{i=1}^{l}\sum_{j=1}^{l}\gamma_{ij}C_{ij}-\varepsilon H(\gamma ),\\ & s.t.\;\Pi (a,b)=\left\{\gamma \in R_{+}^{l\times l}|\gamma 1_{l}=a,\;\gamma^{⊤}1_{l}=b\right\},\\ & C_{ij}={\left\|{\tilde{S}}_{i}^{x}-{\tilde{S}}_{j}^{e}\right\|}_{2}^{2}, \end{array}$$

where $C\in R^{l\times l}$ is the cost matrix using the squared Euclidean distance. $H(\gamma )=\sum_{i,j}\gamma_{ij}(\text{log}\;\gamma_{ij}-1)$ is the entropic regularization term, and ε>0 is a regularization parameter that controls the degree of concentration of the transport plan. $a\in R^{l},b\in R^{l}$ are marginal distributions which are set to be uniform, i.e. $a_{i}=1/l$ and $b_{j}=1/l$ for all *i, j*.

The solution to Equation (6) can be efficiently obtained via the Sinkhorn algorithm (Cuturi 2013). In practice, we employ a numerically stable log-domain variant. The transport matrix can be expressed as:


(7) γ=diag(u)K diag(v),

where K=exp(−C/ϵ) is the kernel matrix, $u\in R^{l}$, $v\in R^{l}$ are scaling vectors iteratively updated to ensure marginal constraints.

#### 2.4.2 Representation mapping

Using the learned transport matrix γ, we compute the cross-modally mapped features as:


(8) $${\tilde{S}}^{x\to e}=\gamma {\tilde{S}}^{e},\;{\tilde{S}}^{e\to x}=\gamma^{⊤}{\tilde{S}}^{x},$$

Here, multiplying by γ projects each modality’s features according to the learned token-level correspondences, yielding cross-modal views ${\tilde{S}}^{x\to e}$ and ${\tilde{S}}^{e\to x}$. These views provide a complementary perspective on regulatory semantics, augmenting the original shared features.

#### 2.4.3 Correspondence-guided gated fusion

To adaptively fuse the original shared features and their cross-modal mapped counterparts, we design a *gating mechanism*.

Specifically, the gate is computed from the concatenated mapped features, enabling the fusion to be conditioned on the reliability of the learned correspondences. The final fused joint representation is obtained by gating-based integration as:


(9) $$\begin{array}{c} g=\sigma \left(W_{2}\phi \left(W_{1}([{\tilde{S}}^{x}\|{\tilde{S}}^{e}])+b_{1}\right)+b_{2}\right),\\ {\tilde{S}}^{\text{fuse}}=g⊙([{\tilde{S}}^{x}\|{\tilde{S}}^{e}])+(1-g)⊙([{\tilde{S}}^{x\to e}\|{\tilde{S}}^{e\to x}]), \end{array}$$

where $g\in {\left[0,1\right]}^{d}$ is a learnable gate that adaptively balances the original and cross-modally mapped features at each feature dimension, $W_{1},W_{2}\in R^{d\times d}$, $b_{1},b_{2}\in R^{d}$ are weighting parameters and biases. σ is sigmoid function, and ϕ is activation function.

The fused representation serves as an intermediate representation for subsequent refinement and fusion. First, it is decomposed along the feature dimension into the *updated modal-wise aligned shared representations*, ${\tilde{S}}^{x}$ and ${\tilde{S}}^{e}$. Second, as a unified shared representation, $\left[S^{x}\|S^{e}\right]$, its features in the central region are *replaced by the fused representation*.

### 2.5 Post-alignment

Building on the semantically aligned representations, we first perform Intra-modal Token Relational Consistency (IRC) to enhance intra-modal consistency, and then aggregate all features for gene expression prediction.

#### 2.5.1 Intra-modal Token Relational Consistency (IRC)

Shared and specific representations encode complementary aspects of the same input sequence. Since the shared components are explicitly refined via cross-modal alignment, they capture more stable token-level relational patterns, while the modality-specific components may exhibit less coherent relational structures among tokens. To maintain intra-modal structural consistency without requiring feature-level similarity, we introduce *Intra-modal Token Relational Consistency* to encourage the relational structure of the specific representations to follow that of the aligned shared space. This regularization is applied within the same TSS-centered region of length *l* where the shared representations are aligned.

For each modality, we compute token-to-token similarity matrices from both the shared and specific representations, which serve as their respective relational distributions. For the DNA sequence modality, these distributions are computed as:


(10) $$Q^{x,s}=\text{softmax}\left({\tilde{S}}^{x}{\left({\tilde{S}}^{x}\right)}^{⊤}\right),\;Q^{x,p}=\text{softmax}\left({\tilde{P}}^{x}{\left({\tilde{P}}^{x}\right)}^{⊤}\right),$$

and for the epigenomic modality:


(11) $$Q^{e,s}=\text{softmax}\left({\tilde{S}}^{e}{\left({\tilde{S}}^{e}\right)}^{⊤}\right),\;Q^{e,p}=\text{softmax}\left({\tilde{P}}^{e}{\left({\tilde{P}}^{e}\right)}^{⊤}\right),$$

Here, $Q^{x,s}$, $Q^{x,p}$, $Q^{e,s}$, and $Q^{e,p}\in R^{l\times l}$, the *i*-th row represents the relational distribution of token *i* with all other tokens. The *Intra-modal Token Relational Consistency* is enforced by minimizing the KL divergence between the relational distributions of the specific and shared representations.


(12) $$L_{\text{IRC}}=\sum_{m\in \{x,e\}}\sum_{i=1}^{l}D_{\text{KL}}(Q_{i}^{m,p}\|Q_{i}^{m,s}),$$

By ensuring that relational patterns in modality-specific components remain coherent with the cross-modally refined structures, this loss enhances intra-modal coherence while preserving discriminative, modality-specific information.

#### 2.5.2 Multimodal fusion and prediction

The goal of multimodal fusion is to integrate complementary regulatory cues and learn a unified representation for prediction.

We first construct a comprehensive multimodal representation by concatenating the modality-specific features (i.e. $P^{x}$ and $P^{e}$) with the shared component (i.e. $S^{x}$ and $S^{e}$).


(13) $$F=[P^{x}\|P^{e}\|S^{x}\|S^{e}],$$

To capture regulatory dependencies across the entire sequence context, we feed the multimodal representation *F* into a two-layer BiMamba. The final gene expression level $\hat{y}\in R$ is then obtained by mean-pooling over the TSS-centered region I, followed by an MLP:


(14) $$\begin{array}{c} \hat{F}=\text{BiMamba}(F),\\ \hat{y}=\text{MLP}\left(\text{MeanPooling}(\{{\hat{F}}_{i}|i\in I\})\right), \end{array}$$

### 2.6 Training objective

Following previous work (Su *et al*. 2025), we adopt the Smooth L1 loss as the main objective function:


(15) $$L_{\text{main}}=\{\begin{array}{cc} \frac{1}{2}{\left(\hat{y}-y\right)}^{2}, & |\hat{y}-y|<1,\\ |\hat{y}-y|-\frac{1}{2}, & \text{otherwise}, \end{array}$$

The composite loss function is:


(16) $$L=L_{\text{main}}+\alpha L_{\text{orth}}+\beta L_{\text{IRC}},$$

where α and β are hyperparameters controlling the strengths of the orthogonal loss and KL divergence loss, respectively.

### 2.7 Complexity analysis

We analyze the computational complexity of the major components of OrchAlign. The Disentanglement (DS) module performs feature decomposition through projection layers with complexity $O(Ld^{2})$, while reducing the dimensionality of the shared representation used for alignment. The Intra-modal Relational Consistency (IRC) module computes token-level relational consistency with complexity $O(l^{2}d)$. The Cross-modal Alignment (CA) module involves focused optimal transport (FOT) within the alignment region. For an alignment window of length *l*, constructing the token-level cost matrix requires $O(l^{2}d)$ complexity, and the Sinkhorn iterations require approximately $O(Tl^{2})$ complexity, where *T* denotes the number of Sinkhorn iterations. Therefore, the overall complexity of CA is $O(l^{2}(d+T))$, exhibiting quadratic scaling with respect to the alignment window length.

## 3 Results

### 3.1 Experimental setup

#### 3.1.1 Task

We predict gene expression levels by modeling Cap Analysis of Gene Expression (CAGE) measurements (Shiraki *et al*. 2003), which provide a quantitative readout of transcription initiation. All CAGE data are obtained from the ENCODE project (ENCODE Project Consortium 2012).

#### 3.1.2 Datasets

Following established studies (Lin *et al*. 2024, Su *et al*. 2025), we focus on three well-characterized cell lines, K562, GM12878 and H1, and aim to predict gene expression levels for 18,377 protein-coding genes. Our model accepts two input modalities: DNA sequence and epigenomic signals. DNA sequences are extracted from the HG38 human reference genome (Schneider and Church 2013). For epigenomic signals, we incorporate two types known to be predictive of CAGE prediction: (1) *DNase-seq* profiles, which indicate chromatin accessibility and highlight open regulatory regions; and (2) *H3K27ac* ChIP–seq signals, a histone modification that serves as a potential marker for active enhancers and promoters. Details of the data sources are provided in the Supplementary Materials, available as supplementary data at *Bioinformatics* online.

#### 3.1.3 Baseline models

We benchmark our model against several well-established baselines, including sequence-only (Enformer (Avsec *et al*. 2021), HyenaDNA (Nguyen *et al*. 2023), Mamba (Gu and Dao 2024), and Caduceus (Schiff *et al*. 2024)), and multimodal models (EPInformer (Lin *et al*. 2024), Caduceus w/signal, Seq2Exp (Su *et al*. 2025)), and AlignMamba (Li *et al*. 2025). Details of the Baseline models are provided in the Supplementary Materials, available as supplementary data at *Bioinformatics* online.

#### 3.1.4 Evaluation metrics

We evaluate all models using three complementary metrics: *Mean Squared Error (MSE)*, which quantifies average squared differences with greater penalty for large errors; *Mean Absolute Error (MAE)*, measuring average absolute deviation for stable accuracy assessment; and *Pearson Correlation (Pearson)*, reflecting linear association and the preservation of relative expression patterns. Together, MSE and MAE assess prediction accuracy, while Pearson evaluates the preservation of relative expression rankings across genes.

#### 3.1.5 Implementation details

We follow the chromosomes-wise data-splitting scheme of Seq2Exp (Su *et al*. 2025): chromosomes 3 and 21 are used for validation, chromosomes 22 and X for testing, and the remaining chromosomes for training.

Both the unimodal encoders and the multimodal fusion module consist of 2 layers, with hidden dimensions of 128 and 256, respectively. Training hyperparameters, including learning rate, batch size, total training steps, optimizer, and scheduler settings, are kept consistent with Seq2Exp to ensure fair comparison. We set our model’s input length to 20 kbp, which retains sufficient genomic context to capture meaningful long-range interactions while significantly lowering computational cost. The TSS-centered region is fixed at 2 kbp, capturing highly informative regulatory signals for gene expression. We also run Seq2Exp with the same input length (20 kbp); this variant is denoted as Seq2Exp w/20k. The reported results are from five runs using random seeds {2, 22, 222, 2222, 22222}, following (Su *et al*. 2025).

### 3.2 Comparison with other baseline methods

We conduct five independent random runs on the K562, GM12878, and H1 cell lines. The mean performance and standard deviations are reported in Table 1, with baseline results directly taken from Seq2Exp for a fair comparison.

**Table 1:** Gene expression prediction results on K562, GM12878 and H1.

Model type	Model	K562			GM12878			H1		
		**MSE** ↓	**MAE** ↓	**Pearson** ↑	**MSE** ↓	**MAE** ↓	**Pearson** ↑	**MSE** ↓	**MAE** ↓	**Pearson** ↑
Sequence-only	Enformer	0.2920 ± 0.0050	0.4056 ± 0.0040	0.7961 ± 0.0019	0.2889 ± 0.0009	0.4185 ± 0.0013	0.8327 ± 0.0025	0.3959 ± 0.0133	0.4591 ± 0.0076	0.4732 ± 0.0151
	HyenaDNA	0.2265 ± 0.0013	0.3497 ± 0.0007	0.8425 ± 0.0008	0.2217 ± 0.0018	0.3562 ± 0.0012	0.8729 ± 0.0010	0.3300 ± 0.0129	0.4130 ± 0.0053	0.5866 ± 0.0178
	Mamba	0.2241 ± 0.0027	0.3416 ± 0.0020	0.8412 ± 0.0021	0.2145 ± 0.0021	0.3446 ± 0.0022	0.8788 ± 0.0011	0.3285 ± 0.0090	0.4100 ± 0.0034	0.5910 ± 0.0050
	Caduceus	0.2197 ± 0.0038	0.3327 ± 0.0070	0.8475 ± 0.0014	0.2124 ± 0.0037	0.3436 ± 0.0031	0.8819 ± 0.0009	0.3179 ± 0.0081	0.4054 ± 0.0045	0.6035 ± 0.0064
Multimodal	Caduceus w/sig	0.1959 ± 0.0036	0.3187 ± 0.0036	0.8630 ± 0.0008	0.1942 ± 0.0058	0.3269 ± 0.0048	0.8928 ± 0.0017	0.2722 ± 0.0082	0.3764 ± 0.0034	0.6714 ± 0.0113
	EPInformer	0.2140 ± 0.0042	0.3291 ± 0.0031	0.8473 ± 0.0017	0.1975 ± 0.0031	0.3246 ± 0.0025	0.8907 ± 0.0011	0.3061 ± 0.0074	0.4077 ± 0.0057	0.6201 ± 0.0124
	AlignMamba	0.1965 ± 0.0053	0.3147 ± 0.0035	0.8604 ± 0.0034	0.2089 ± 0.0032	0.3311 ± 0.0059	0.8799 ± 0.0019	0.2535 ± 0.0075	0.3722 ± 0.0093	0.6970 ± 0.0075
	Seq2Exp	0.1856 ± 0.0024	0.3054 ± 0.0032	0.8723 ± 0.0015	0.1873 ± 0.0016	0.3137 ± 0.0023	0.8951 ± 0.0013	0.2628 ± 0.0095	0.3756 ± 0.0072	0.6825 ± 0.0139
	Seq2Exp w/20k	0.1893 ± 0.0058	0.3127 ± 0.0054	0.8680 ± 0.0022	0.1850 ± 0.0106	0.3162 ± 0.0095	0.8965 ± 0.0054	0.2657 ± 0.0084	0.3783 ± 0.0077	0.6768 ± 0.0112
	**OrchAlign**	**0.1727 ± 0.0027**	**0.2938 ± 0.0052**	**0.8781 ± 0.0021**	**0.1662 ± 0.0067**	**0.2970 ± 0.0075**	**0.9063 ± 0.0021**	**0.2367 ± 0.0056**	**0.3566 ± 0.0053**	**0.7171 ± 0.0072**
	Improv.	6.95%	3.80%	0.66%	10.16%	5.32%	1.08%	6.63%	1.56%	2.01%

Best results are in **bold**, second best are underlined.

We have several key observations: (1) *Our proposed OrchAlignconsistently outperforms all baselines across cell lines and evaluation metrics*, achieving MSE reductions of up to 6.95%, 10.16% and 6.63% over the strongest multimodal baseline on K562, GM12878 and H1, respectively. These gains can be attributed to the proposed three-stage design: encoders and disentanglement yield purified shared representations for alignment, focused OT establishes sparse and precise token-level correspondences, and post-alignment refinement and fusion enhance intra-modal coherence and enable effective multimodal fusion. (2) *The improvement in Pearson correlation indicates that OrchAlign better preserves the* relative expression levels *across genes*. This implies that our alignment mechanism more faithfully learns the regulatory logic governing transcriptional differences. The concurrent gains in both ranking (Pearson) and absolute error (MSE/MAE) demonstrate *a comprehensive advance in prediction quality*. (3) On the challenging K562 cell line, characterized by pronounced regulatory heterogeneity, *OrchAlign* delivers substantial improvement over baselines, *highlighting its strong robustness in modeling complex regulatory variation*. This likely stems from its FOT alignment, which adaptively learns cross-modal correspondences for each gene, thereby effectively capturing their individual regulatory patterns. (4) *Multmodal models consistently outperform sequence-only models across all evaluated cell lines and metrics*, indicating the critical and complementary role of epigenomic information in gene expression prediction. For example, the Caduceus model augmented with epigenomic signals (i.e. Caduceus w/signals) achieves a significant reduction in MSE of approximately 8%–10% compared to its sequence-only version on two cell lines. (5) Compared with AlignMamba, which also employs OT-based multimodal alignment, *OrchAlign* achieves consistent improvements across cell lines, highlighting the effectiveness of our alignment framework for DNA–epigenomic regulatory modeling. (6) Among sequence-only models, Caduceus achieves the strongest performance, demonstrating the advantage of its bidirectional Mamba in capturing regulatory signals from upstream and downstream. *Our method extends Caduceus with explicit cross-modal alignment and fusion*, yielding consistent and substantial improvements over all baselines in gene expression prediction.

### 3.3 Ablation study

To evaluate the contribution of each component in *OrchAlign*, we conduct ablation studies using a stepwise module addition strategy. We start from a base model (first row of Table 2) that excludes all proposed components (Cross-modal Alignment (CA), Disentanglement (DS), and Intra-modal Relational Consistency (IRC)). The base model independently encodes each modality and fuses them via simple concatenation. We incrementally add CA, DS, and IRC to assess their individual contributions.

**Table 2:** Ablation study of *OrchAlign*.

Modules			K562			GM12878			H1		
CA	DS	IRC	**MSE** ↓	**MAE** ↓	**Pearson** ↑	**MSE** ↓	**MAE** ↓	**Pearson** ↑	**MSE** ↓	**MAE** ↓	**Pearson** ↑
×	×	×	0.1972	0.3121	0.8604	0.2084	0.3330	0.8857	0.2627	0.3782	0.6799
✓	×	×	0.1765	0.2990	0.8759	0.1782	0.3038	0.9006	0.2415	0.3620	0.7109
✓	✓	×	0.1749	0.2949	**0.8784**	0.1690	0.2985	0.9011	0.2375	0.3587	0.7166
✓	✓	✓	**0.1727**	**0.2938**	0.8781	**0.1662**	**0.2970**	**0.9063**	**0.2367**	**0.3566**	**0.7171**

CA, Cross-modal alignment; DS, Disentanglement; IRC, Intra-modal Relational Consistency. Best results are in **bold**.

Several key observations can be drawn from Table 2. First, *the CA module contributes the most to performance gains*. Introducing CA alone (row 2) reduces MSE by approximately 10.5% in K562, 14.5% in GM12878, and 8.1% in H1 compared to the base model, demonstrating the foundational role of CA alignment in establishing reliable cross-modal correspondences. Second, *Disentanglement further improves performance.* Adding DS to CA (row 3) yields an additional MSE reduction of about 0.9% in K562, 5.1% in GM12878, and 1.7% in H1 over the CA-only setup. This demonstrates that aligning in a purified shared subspace is more effective than aligning the original, entangled representations. Finally, *Intra-modal Relational Consistency refines modality-specific representations.* Incorporating IRC (row 4, i.e. *OrchAlign*) achieves the best overall results, further reducing MSE over the CA+DS by aligning the relational structures of specific components with the shared aligned space.

#### 3.3.1 Cell-specific analysis

CA delivers the strongest and most consistent gain in both cell lines, confirming its foundational role in cross-modal alignment. The benefits of DS and IRC, however, vary with cellular context. In GM12878, which exhibits more stable chromatin states and more separable regulatory factors, both DS and IRC yield clear additional gains. In K562 and H1, by contrast, the regulatory landscape is more complex and dynamic, making factor separation inherently more difficult. Correspondingly, the improvements from DS and IRC are more modest in this setting. Nevertheless, the complete *OrchAlign* (CA+DS+IRC) achieves the best performance in both settings, demonstrating its ability to adapt effectively across distinct biological environments.

#### 3.3.2 Computational cost of model configurations

We report the training time per epoch and GPU memory usage of different module configurations in Table 3. The CA module introduces the largest overhead due to the optimal transport operation, whereas DS and IRC introduce only modest additional computational and memory costs. These observations are consistent with the theoretical complexity analysis in Section 2.7, where the computational complexity of CA incorporates the iterative OT procedure.

**Table 3:** Computational cost of different model configurations.

Configuration	Training time (min)	GPU memory (GB)
Base	29	13.8
+ CA	43	39.6
+ CA + DS	41	40.8
*OrchAlign*	46	42.2

### 3.4 Performance on extreme expression genes

To further assess whether our model maintains predictive advantages beyond average expression patterns, we performed an additional analysis of genes with extreme expression levels (top and bottom 10%) in K562 and GM12878. As shown in Fig. 2, *OrchAlign* consistently outperforms Seq2Exp on both subsets. For high-expression genes, our model reduces MSE by approximately 10% on K562 and 7% on GM12878 relative to Seq2Exp. For low-expression genes, the improvements are more pronounced, with MSE reductions of nearly 20% on K562 and 37% on GM12878. These results demonstrate that our model is particularly effective on extreme expression genes and does not rely solely on average expression patterns.

**Figure 2:**
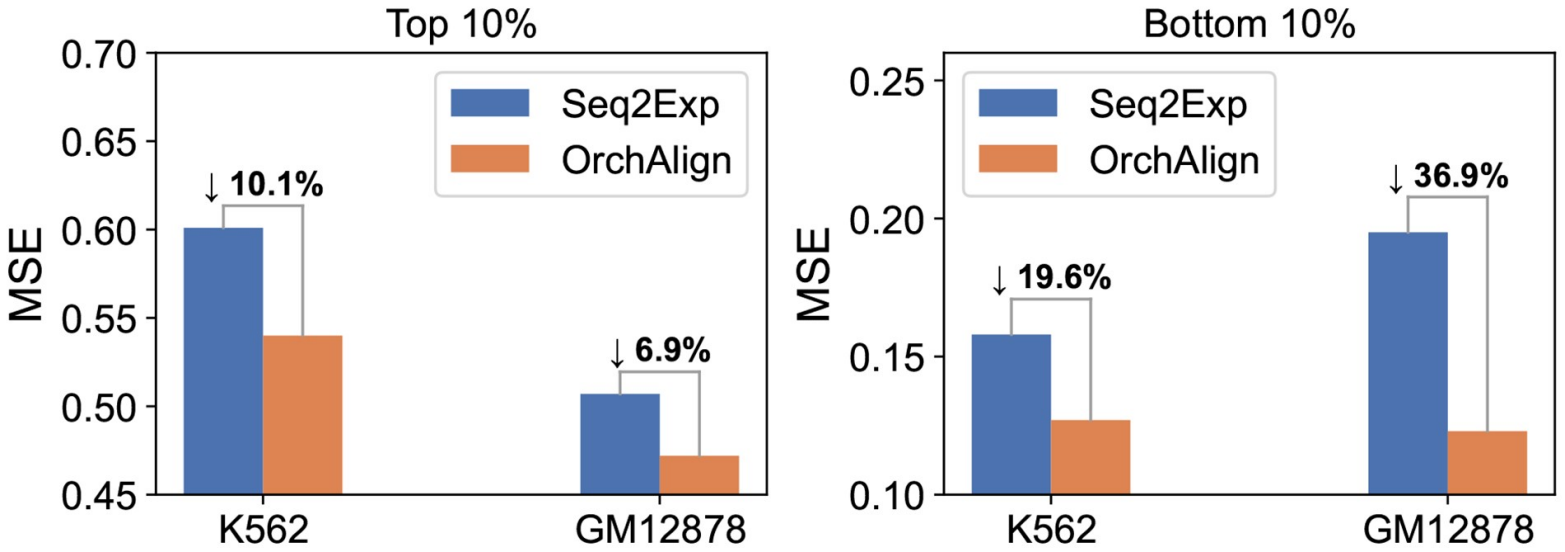
MSE comparison between Seq2Exp and OrchAlign on extreme expression genes (top and bottom 10% ranked by expression level).

### 3.5 Case study

To examine how the alignment design in *OrchAlign* captures biologically meaningful cross-modal correspondences, we conduct a case study using the CA+DS configuration and compare it with the base model without explicit alignment. We analyze the gene *MAPK1* in the K562 cell line to illustrate the learned DNA-to-epigenomic alignment. *MAPK1* is a representative regulatory gene with well-characterized H3K27ac peaks, facilitating intuitive visualization of alignment patterns.

For fair comparison, we extract representations within a central 2  kb window from both models, including the outputs of unimodal encoders for the base model and the aligned shared representations produced by the cross-modal alignment module for the CA+DS model. The optimal transport algorithm is then applied to these representations to compute transport matrices and Wasserstein distances (WD). We compare visualized transport matrices (Fig. 3A and B) with the true H3K27ac signal profile (Fig. 3C) and evaluate their metrics (Fig. 3D) (MSE for predictive performance and WD for modality discrepancy). Due to the extreme sparsity of the transport matrices, a small constant (1e-12) is added before log-normalization to enable visualization.

**Figure 3:**
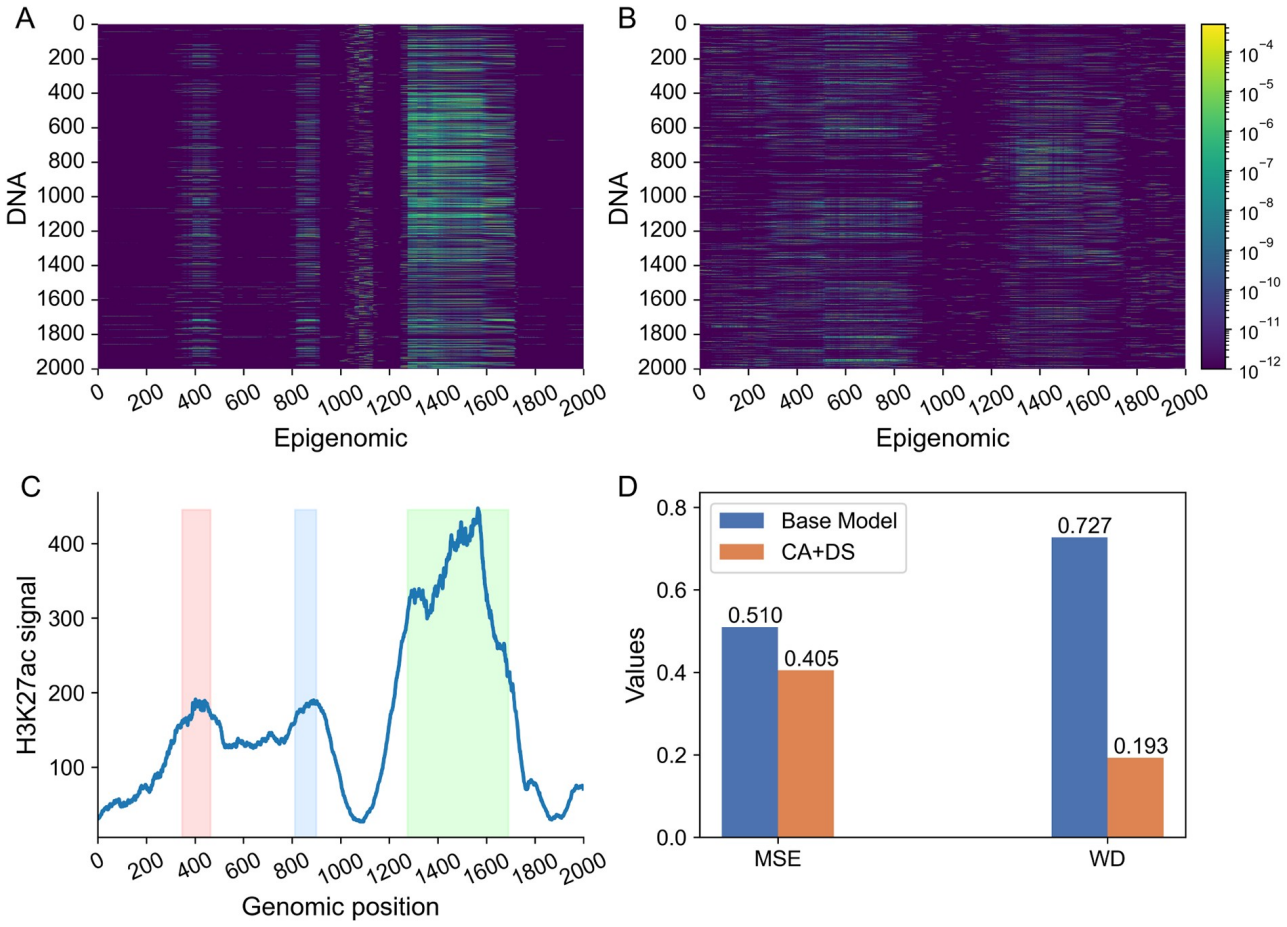
Optimal transport matrix and metrics on the *MAPK1* gene. (A) Optimal transport matrix with alignment (CA+DS). (B) Optimal transport matrix without alignment (base model). (C) H3K27ac signal across the genomic region. (D) Comparison of MSE and Wasserstein distance between the two models.

The proposed FOT-based cross-modal alignment improves biological interpretability. Specifically, the transport matrix with the CA model exhibits sparse and focused correspondences (Fig. 3A): DNA tokens align preferentially with epigenomic positions near active H3K27ac regions, concentrating transport mass around known regulatory peaks (the shaded peak regions in Fig. 3C). This pattern indicates that FOT-based CA captures biologically relevant associations between sequence features and epigenomic activity. In contrast, the matrix with base model (Fig. 3B) is more diffuse, lacking a clear focus on regulatory hotspots.

Moreover, these qualitative observations are further supported by quantitative metrics (Fig. 3D). Compared to the base model, CA+DS significantly reduces both the MSE (by 21.6%) and the Wasserstein distance (by 73.5%), indicating improved predictive accuracy and alignment quality.

Together, these results show that FOT-based cross-modal alignment enhances numerical performance while yielding biologically coherent and interpretable cross-modal correspondences.

To complement the strong H3K27ac signal case of *MAPK1*, we further analyzed *PAGE1*, a gene with substantially weaker proximal H3K27ac signals (Fig. 4C). Compared with *MAPK1*, *PAGE1* exhibits a less concentrated transport pattern (Fig. 4A), suggesting increased alignment uncertainty under weak epigenomic signals. This observation is further supported by the test set analysis, where low-H3K27ac genes show higher OT entropy than high-H3K27ac genes (Fig. 4B). Despite the reduced alignment concentration, OrchAlign still improves expression prediction over Seq2Exp (Fig. 4D). Moreover, high-H3K27ac genes generally achieve larger improvements, likely because stronger epigenomic signals provides more discriminative cues for cross-modal alignment.

**Figure 4:**
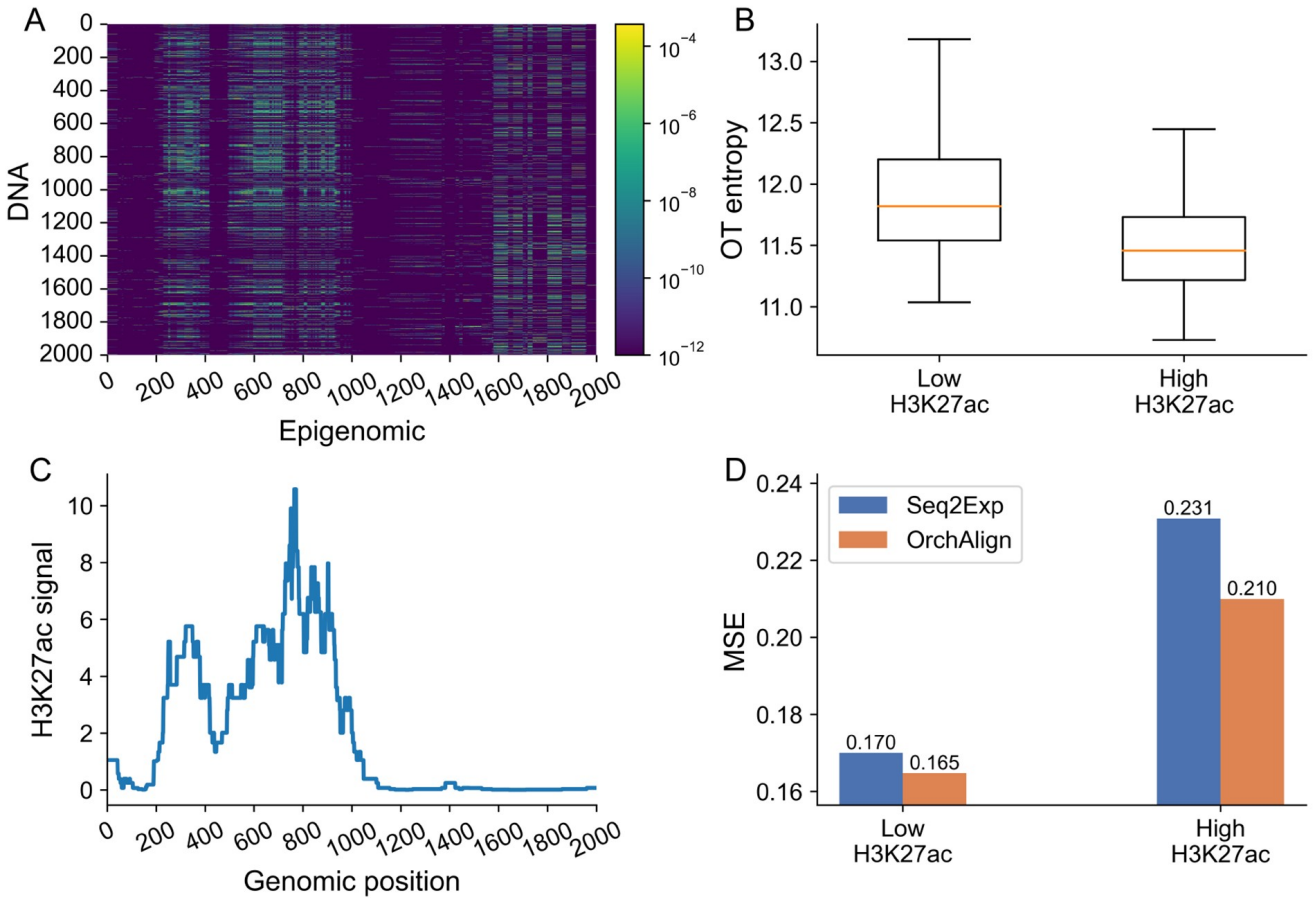
Cross-modal alignment under different signal strengths. (A) Optimal transport matrix of the *PAGE1* gene with relatively weak H3K27ac signals. (B) Comparison of OT entropy between genes with low and high H3K27ac signals in the test set. (C) H3K27ac signal across the genomic region. (D) MSE comparison between OrchAlign and Seq2exp. Low- and high-H3K27ac groups are defined as genes with bottom and top quartiles of proximal H3K27ac signal levels, respectively.

### 3.6 Parameter sensitivity analyses

*OrchAlign* involves several key hyperparameters, including *l*, ϵ, α, and β. We evaluated their sensitivity on K562 and GM12878 (Figs.  5, 7–9). For each hyperparameter setting, the reported metrics were averaged across all genes in the test set. Sensitivity results on H1 are provided in the Supplementary Materials, available as supplementary data at *Bioinformatics* online.

#### 3.6.1 Alignment window size *l*

The alignment window size *l* determines the genomic region considered for cross-modal alignment. To investigate its impact, we compared five settings: 0.5 kb, 1 kb, 2 kb (default), 3 kb, and 4 kb, and the performance results are shown in Fig. 5. Both smaller and larger windows degrade performance relative to the default setting. The inferior performance of the smaller window can be attributed to missing key proximal regulatory signals that reside beyond this narrow region. Conversely, the larger window likely incorporates irrelevant distal signals, thereby weakening the cross-modal alignment quality. Expanding the alignment window also substantially increases computational costs due to the quadratic complexity of the optimal transport operation (Fig. 6). Therefore, we adopt the 2 kb alignment window as the default setting.

**Figure 5:**
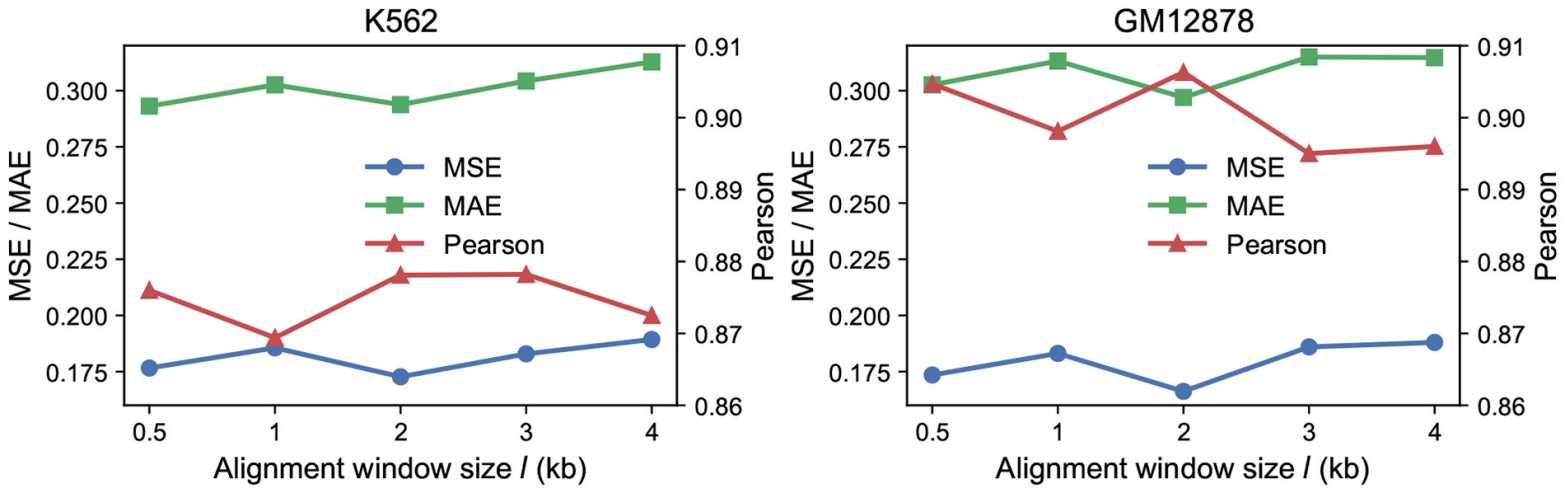
Performance w.r.t. different alignment window sizes.

**Figure 6:**
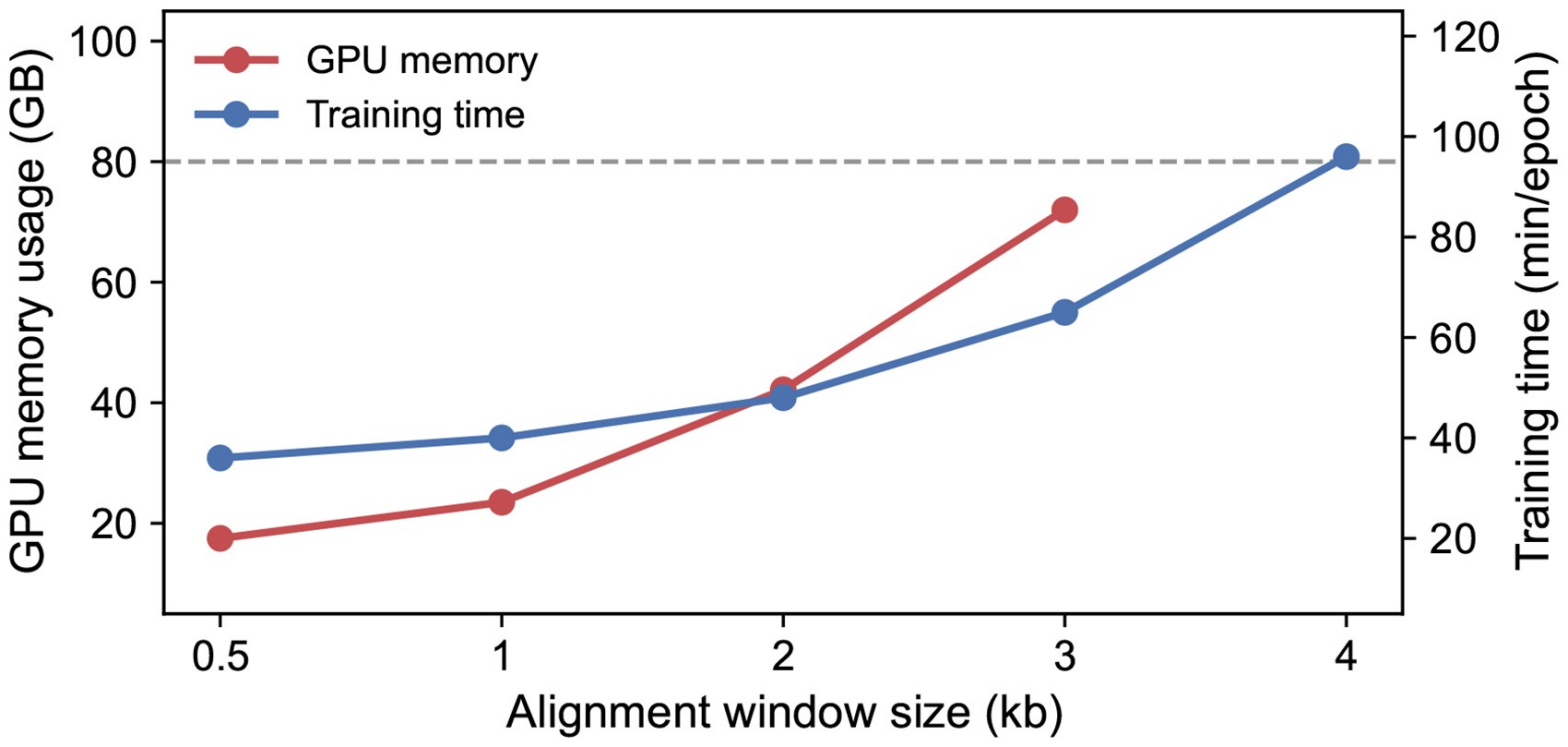
Computational cost under different alignment window sizes. Both GPU memory consumption and training time increase with the alignment window size. The 4 kb window exceeds the 80 GB memory capacity of a single GPU, and its training time was obtained with gradient accumulation.

#### 3.6.2 Entropic regularization ϵ

This parameter trades off alignment sharpness and stability. Lower values promote sharper, more deterministic transport plans, while higher values encourage smoother, more stable alignments. We performed cell-specific tuning due to differing regulatory complexity (Fig. 7). K562 achieved optimal performance at ϵ=0.05, favoring the stability of smoother alignments in its complex regulatory landscape. In contrast, GM12878 performed best with the sharper alignment of ϵ=0.01, reflecting its more stable chromatin environment. This underscores the need to adapt ϵ to the biological context.

**Figure 7:**
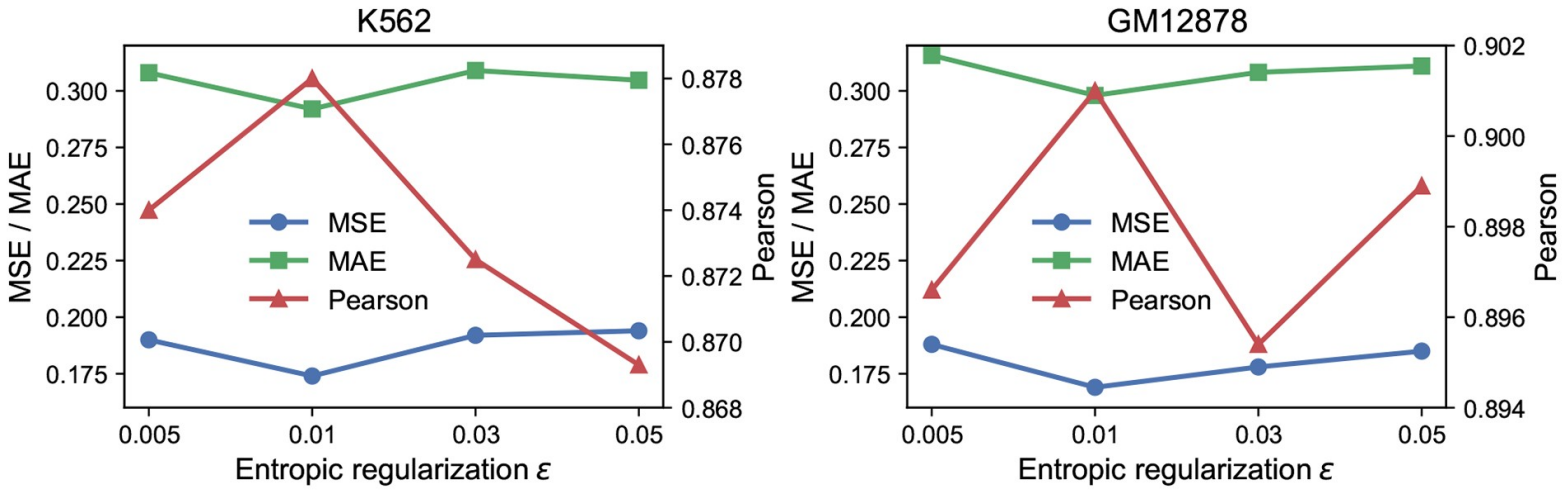
Performance w.r.t. different ϵ.

#### 3.6.3 Orthogonal constraint α

The weight controls the strength of the orthogonal constraint that separates shared and modality-specific representations. Candidate values {0.01,0.02,0.03,0.04} were evaluated. We observe that α=0.02 delivers the best performance on both cell lines (Fig. 8). The performance variation across α reflects a trade-off between disentanglement sufficiency and representational flexibility.

**Figure 8:**
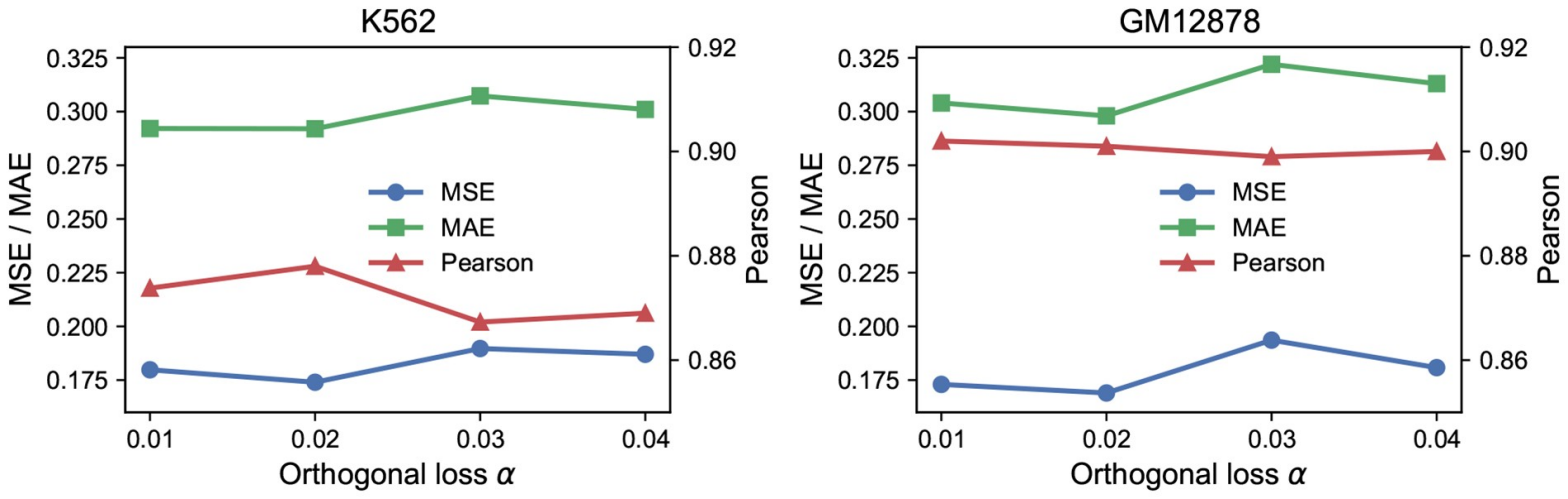
Performance w.r.t. different α.

#### 3.6.4 KL divergence ***β***

This coefficient governs the strength of intra-modal relational consistency. The candidate values of β are from {0.001,0.003,0.005,0.007}. K562 and GM12878 achieve their best performance at β=0.005 and β=0.001, respectively (Fig. 9). Larger values may overly constrain specific representations and suppress unique information, whereas smaller values provide insufficient structural regularization, potentially degrading performance. The effect significantly varies in different cells; K562 exhibits greater sensitivity to β than GM12878, which aligns with its more complex and noisy regulatory landscape.

**Figure 9:**
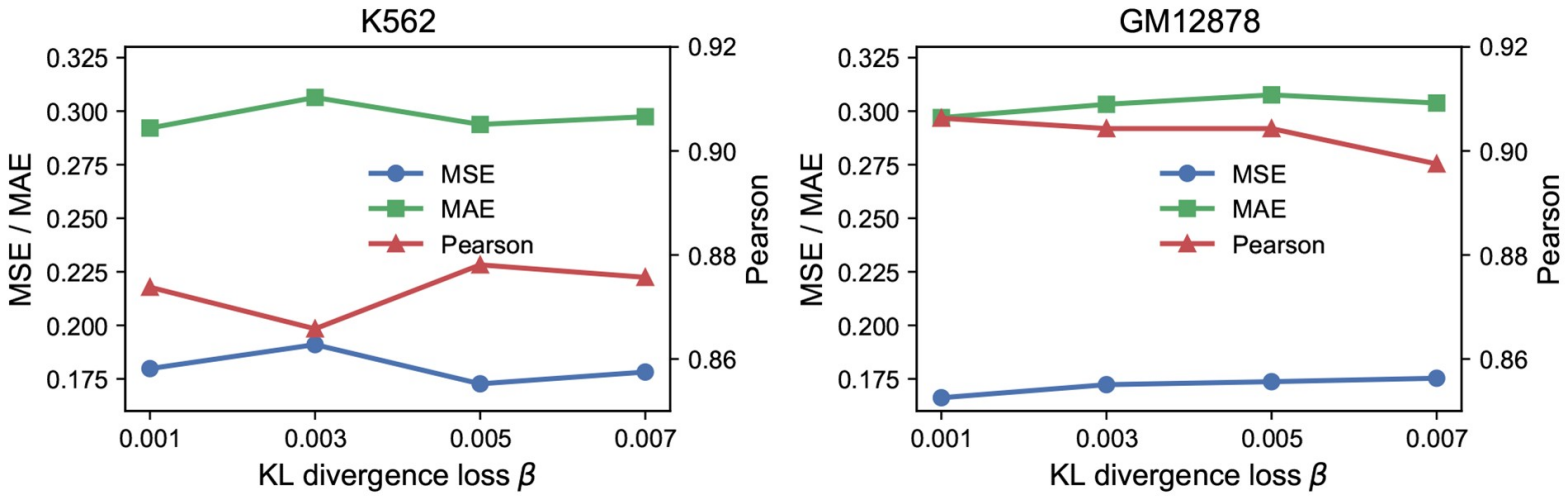
Performance w.r.t. different β.

## 4 Discussion

We introduce *OrchAlign* to address the challenge of aligning heterogeneous genomic modalities (e.g. DNA sequences and epigenomic signals) for gene expression prediction. OrchAlign combines modality disentanglement, focused entropic-regularized optimal transport, and intra-modal relational consistency to capture token-level regulatory correspondence between DNA sequences and epigenomic signals while preserving modality-specific information. Extensive experiments on three cell lines demonstrate that *OrchAlign* consistently outperforms strong baselines in gene expression prediction performance, while producing more coherent cross-modal alignments. These results highlight the importance of explicitly modeling both cross-modal correspondence and intra-modal structural preservation in heterogeneous genomic integration. Overall, this approach provides a unified framework for heterogeneous genomic data fusion, suggesting a promising direction for advancing multimodal learning in functional genomics. Future work may explore how multimodal information from broader genomic regions can be more effectively utilized, where abundant background signals make informative cross-modal collaboration more challenging.

## Supplementary Material

btag711_Supplementary_Data

## Data Availability

Data are available in the online supplementary material. Code is available at GitHub (https://github.com/XMUDM/OrchAlign) and Zenodo (https://doi.org/10.5281/zenodo.21936202).
