# Supplementary material for: OrchAlign: orchestrated multimodal alignment for gene expression prediction
Source: Bioinformatics. 2026 Sep 29;42(10):btag711. doi: 10.1093/bioinformatics/btag711 (PMC13661263; doi:10.1093/bioinformatics/btag711)
Supplement: Supplementary file 1 [file btag711_supplementary_data.pdf]

Supplementary Material

Supplementary related work

Gene expression prediction

Deep learning approaches have become the dominant paradigm for gene expression prediction, including convolutional neural networks (CNNs) (Kelley 2020; Kelley et al. 2018), Transformers (Avsec et al. 2021), and graph neural networks (GNNs) (Karbalayghareh et al. 2022). Enformer (Avsec et al. 2021) employs Transformer architectures to explicitly model distal regulatory interactions within DNA sequences. Recent approaches, such as EPInformer (Lin et al. 2024) and Seq2Exp (Su et al. 2025), jointly leverage DNA sequences and epigenomic signals to improve expression prediction. Nevertheless, these methods typically integrate DNA sequences and epigenomic signals via feature concatenation or summation, lacking explicit cross-modal alignment to establish fine-grained correspondence between different modalities.

Optimal transport-based cross-modal alignment

Cross-modal alignment aims to establish correspondence between representations from different modalities. Optimal transport (OT) provides a principled framework for learning such cross-modal correspondence by matching modality distributions under global constraints. OT has been widely explored for cross-modal integration in various domains, including single-cell multi-omics (Demetci et al. 2022b; Gossi et al. 2023; Demetci et al. 2022a) and computer vision applications (Li et al. 2025). For example, SCOT and SCOTv2 (Demetci et al. 2022b,a) apply OT to identify correspondences between cells from different single-cell modalities, performing cell-level multimodal integration. Recent OT-based multimodal alignment methods in computer vision, such as AlignMamba (Li et al. 2025), explore fine-grained correspondence learning between heterogeneous modalities. However, existing approaches are not designed to model correspondence between genomic sequences and epigenomic signals.

Supplementary results

Experimental details

Model and training settings

The complete model configuration and training hyperparameters used in our experiments are summarized in Table S1.

Data processing

Gene expression exhibits substantial cell-type specificity. Following prior studies, we conduct experiments on three well-characterized human cell lines, K562, GM12878 and H1.

CAGE. Cap Analysis of Gene Expression (CAGE) quantifies transcription initiation activity at transcription start sites (TSS), providing a genome-wide measurement of gene expression levels. Each gene is associated with a CAGE signal representing its expression level. In this study, CAGE data are obtained from the FANTOM5 project (Andersson et al. 2014) for the K562 (CNhs11250), GM12878 (CNhs12333), and H1 (CNhs13964) cell lines. The CAGE assay is based on high-throughput sequencing of 5' ends of capped RNA molecules, enabling precise mapping of transcription start sites (Carninci et al. 2006).

DNase-seq. DNase-seq (DNase I hypersensitive site sequencing) identifies regions of open chromatin by measuring DNase I cleavage signals, which reflect chromatin accessibility. Higher signal values

Table S1: Model configuration and training hyperparameters used in our experiments.

Setting	Values
Encoder layers	2
Fusion layers	2
Encoder dimension	128
Fusion dimension	256
Training steps	50000
Batch size	8
Learning rate	5×10^{-4}
Scheduler strategy	Cosine decay with linear warmup
Optimizer	AdamW
Optimizer betas	$\beta_1, \beta_2 = 0.9, 0.95$

indicate regions more accessible to regulatory proteins, such as promoters, enhancers, and transcription factor binding sites. We obtained DNase-seq data from the ENCODE project (Consortium et al. 2012) for K562 (ENCFF414OGC), GM12878 (ENCFF960FMM), and H1 (ENCFF232GUZ) directly in bigWig format, which provides genome-wide signal profiles.

H3K27ac. H3K27ac denotes histone H3 lysine 27 acetylation, a histone modification associated with active promoters and enhancers. Elevated H3K27ac signals indicate regulatory activity and potential gene activation. The data were also obtained from the ENCODE project (Consortium et al. 2012) for K562 (ENCFF465GBD), GM12878 (ENCFF798KYP), and H1 (ENCFF771GNB), in bigWig format providing genome-wide signal profiles.

Baseline models

We benchmark our model against several well-established baselines, including sequence-only (Enformer, HyenaDNA, Mamba, and Caduceus), and multimodal models (EPInformer, Caduceus w/signal and Seq2Exp).

Enformer (Avsec et al. 2021): a CNN-Transformer hybrid architecture to predict gene expression from long-range DNA sequences.

HyenaDNA (Nguyen et al. 2023): an efficient DNA foundation model built on the Hyena operator, designed for long-range genomic sequence modeling.

Mamba (Gu and Dao 2024): an advanced sequence model with linear-time complexity that incorporates a selective mechanism.

Caduceus (Schiff et al. 2024): a bidirectional and reverse-complement-equivariant variant of Mamba, optimized for DNA sequence modeling.

Caduceus w/signal: an extension of Caduceus that jointly encodes DNA sequences and epigenomic signals.

EPInformer (Lin et al. 2024): a recently proposed model that extends the Activity-By-Contact (ABC) framework (Fulco et al. 2019) for gene expression prediction. It uses DNase-seq peaks to define candidate regulatory regions and employs an attention mechanism to aggregate enhancer signals.

Seq2Exp (Su et al. 2025): a state-of-the-art model that learns regulatory element masks to identify influential regions for gene expression prediction.

AlignMamba (Li et al. 2025): a multimodal Mamba framework originally proposed for vision-language tasks. We adapt it to genomic multimodal gene expression prediction while preserving its OT-based local alignment and MMD (Maximum Mean Discrepancy)-based global alignment.

Parameter sensitivity analysis on the H1 cell line

Following the same settings as the main experiments, we evaluated the sensitivity of four key hyperparameters (l, ϵ, α, and β) on the H1 cell line. The results are summarized in Fig. S1. The 2 kb central window achieves the best performance, further supporting the use of focused alignment around the transcription start site. The optimal orthogonal constraint weight ($\alpha = 0.02$) remains consistent across the evaluated cell lines. The optimal values of the OT entropic regularization coefficient ϵ vary across cell lines, with H1 achieving the best performance at $\epsilon = 0.03$, between the optimal values observed for K562 (0.05) and GM12878 (0.01). This variation indicates that the preferred level of

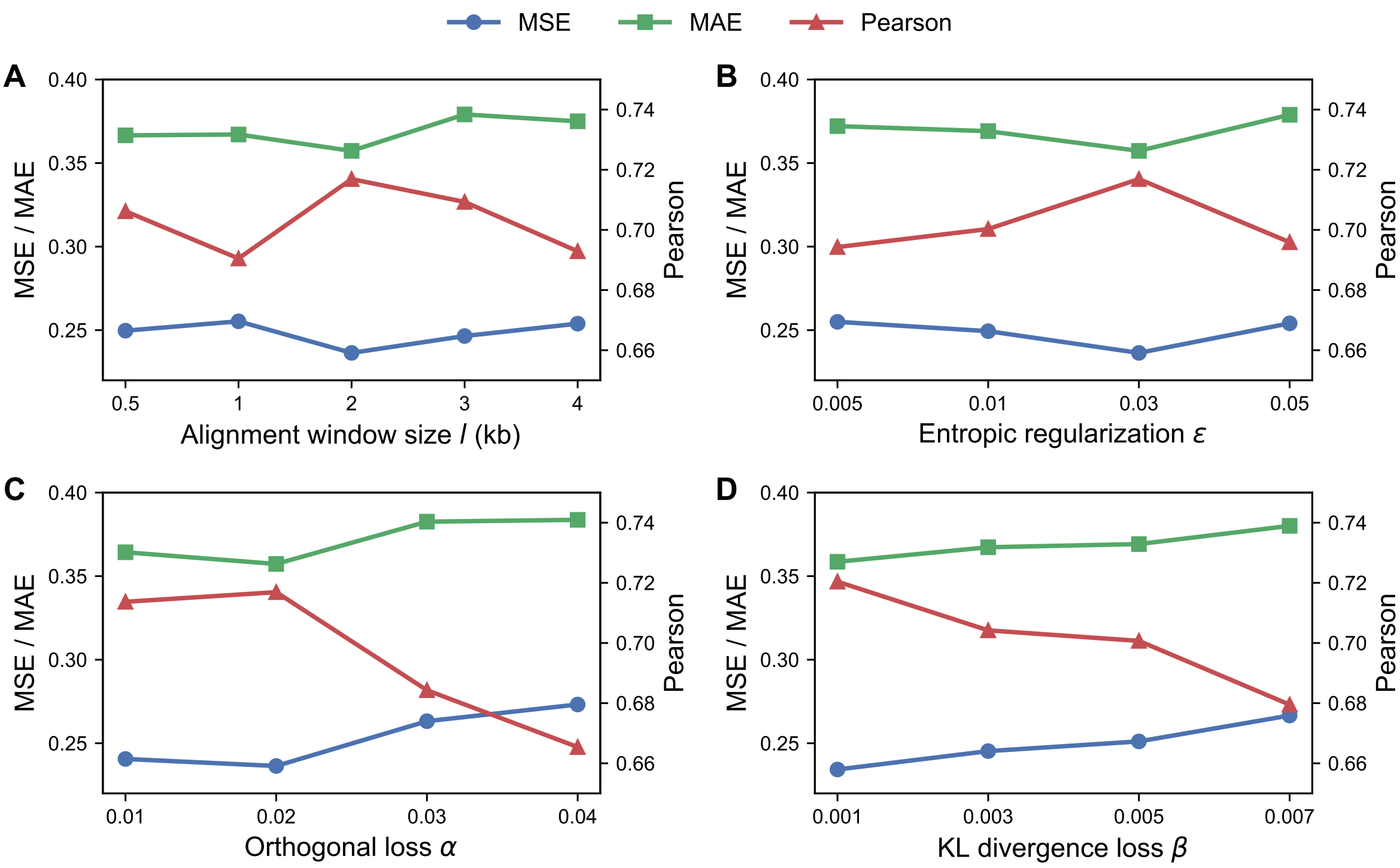

Fig. S1: (A) Performance w.r.t. different l. (B) Performance w.r.t. different ϵ. (C) Performance w.r.t. different α. (D) Performance w.r.t. different β.

entropic regularization may differ across cell lines. For the KL divergence coefficient β, H1 achieves the best performance at $\beta = 0.001$, which is consistent with the optimal value observed in GM12878.

References

Andersson R, Gebhard C, Miguel-Escalada I, Hoof I, Bornholdt J, Boyd M, Chen Y, Zhao X, Schmidl C, Suzuki T, et al. 2014. An atlas of active enhancers across human cell types and tissues. Nature 507(7493): 455–461

Avsec Ž, Agarwal V, Visentin D, Ledsam JR, Grabska-Barwinska A, Taylor KR, Assael Y, Jumper J, Kohli P, Kelley DR. 2021. Effective gene expression prediction from sequence by integrating long-range interactions. Nature methods 18(10): 1196–1203

Carninci P, Sandelin A, Lenhard B, et al. 2006. Genome-wide analysis of mammalian promoter architecture and evolution. Nature Genetics 38(6): 626–635

Consortium EP, et al. 2012. An integrated encyclopedia of dna elements in the human genome. Nature 489(7414): 57

Demetci P, Santorella R, Chakravarthy M, Sandstede B, Singh R. 2022a. Scotv2: Single-cell multi-omic alignment with disproportionate cell-type representation. Journal of Computational Biology 29(11): 1213–1228

Demetci P, Santorella R, Sandstede B, Noble WS, Singh R. 2022b. Scot: single-cell multi-omics alignment with optimal transport. Journal of computational biology 29(1): 3–18

Fulco CP, Nasser J, Jones TR, Munson G, Bergman DT, Subramanian V, Grossman SR, Anyoha R, Doughty BR, Patwardhan TA, et al. 2019. Activity-by-contact model of enhancer–promoter regulation from thousands of crispr perturbations. Nature genetics 51(12): 1664–1669

Gossi F, Pati P, Chouvardas P, Martinelli AL, Kruithof-de Julio M, Rapsomaniki MA. 2023. Matching single cells across modalities with contrastive learning and optimal transport. Briefings in

bioinformatics 24(3): bbad130

Gu A, Dao T. 2024. Mamba: Linear-time sequence modeling with selective state spaces. In: First conference on language modeling

Karbalayghareh A, Sahin M, Leslie CS. 2022. Chromatin interaction–aware gene regulatory modeling with graph attention networks. Genome Research 32(5): 930–944

Kelley DR. 2020. Cross-species regulatory sequence activity prediction. PLoS computational biology 16(7): e1008050

Kelley DR, Reshef YA, Bileschi M, Belanger D, McLean CY, Snoek J. 2018. Sequential regulatory activity prediction across chromosomes with convolutional neural networks. Genome research 28(5): 739–750

Li Y, Xing Y, Lan X, Li X, Chen H, Jiang D. 2025. Alignmamba: Enhancing multimodal mamba with local and global cross-modal alignment. In: Proceedings of the Computer Vision and Pattern Recognition Conference, pp 24774–24784

Lin J, Luo R, Pinello L. 2024. Epinformer: a scalable deep learning framework for gene expression prediction by integrating promoter-enhancer sequences with multimodal epigenomic data. bioRxiv

Nguyen E, Poli M, Faizi M, Thomas A, Wornow M, Birch-Sykes C, Massaroli S, Patel A, Rabideau C, Bengio Y, et al. 2023. Hyenadna: Long-range genomic sequence modeling at single nucleotide resolution. Advances in neural information processing systems 36: 43177–43201

Schiff Y, Kao CH, Gokaslan A, Dao T, Gu A, Kuleshov V. 2024. Caduceus: Bi-directional equivariant long-range dna sequence modeling. Proceedings of machine learning research 235: 43632

Su X, Yu H, Zhi D, Ji S. 2025. Learning to discover regulatory elements for gene expression prediction. In: The Thirteenth International Conference on Learning Representations, ICLR 2025, Singapore, April 24-28, 2025. OpenReview.net, URL https://openreview.net/forum?id=Mfnh1Sqdwf
